# Headache Medicine Grand Challenge: Headache: A New Frontier, A New Challenge

**DOI:** 10.3389/fpain.2021.690683

**Published:** 2021-08-09

**Authors:** Frederick G. Freitag

**Affiliations:** Department of Neurology, Medical College of Wisconsin, Milwaukee, WI, United States

**Keywords:** migraine, rational pharmacology, genetic loci, gut biome, neuroimaging, receptor modeling

## Introduction

Growing into adulthood, I was raised on “Star Trek.” I was entranced by the idea of exploring new frontiers, going where no man has gone before. Headache Medicine is like “Star Trek” in that regard. We are exploring new ideas, new frontiers across a spectrum of ideas, and in many respects going where our predecessors might only have imagined. To explore a new frontier implies that one started from someplace well known, perhaps familiar.

Headaches are as old as humanity. We have evidence of trephined skulls, and from a specimen I saw at the International Museum of Surgical Science years ago, there was evidence of bone healing. The Assyrians (1) wrote of migraine, and the Jewish Proverbs (2) told us to avoid young red wines to avoid migraines. In the third Century BCE, Greek Hippocrates (3) offered clues to the diagnosis. They used the term ημ*ι*κρᾱνíα^1^. This term was eventually transliterated across languages to become “migraine.”

We progressed in our understanding of migraines' physical nature and the humors of antiquarian medicine to a “neurological storm” (4) by the late 1800s. Wolff's view (5) of the vascular nature of migraines grew from his experiences. It began from the nature of the pain description of migraine. Later, his years as a flight surgeon during World War II and pilot's stories of treating their migraines in flight added to his interest. After the war, the discovery, use of, and research on ergotamine (6) paved the way for investigations. This culminated in his work with Ray and Wolff (7) exploring pain-sensitive structures inside the cranium and examining migraine patients' extracranial and intracranial response to ergotamine. It was supported by biochemical evidence in the ‘70s (8) until Lance (9) proposed a new neuro-vascular basis for migraine regulated in the Trigeminal Nucleus, Posterior Aqueductal Gray, Red Nuclei producing the Hypothalamic, Cortical, and vascular effects seen with the methods available in migraine attacks.

The focus on migraine among the more than 300 causes of headache is natural. Essentially, all people will experience what may be termed a tension headache in their lifetime. However, given the large (up to 40%) population who will at some time experience the severity of a migraine, with its impact on quality of life and productivity, and attendant direct and indirect costs, the preeminence of the focus on migraine in the world of headache medicine is not surprising.

The prevalence of Migraines is roughly the same worldwide (10), across ethnic groups and access to financial resources, nutrition, work, and environment. The only significant variables are genetic determinants of sex and age.

The severity and significance of migraine's clinical features led the World Health Organization to classify it among the top 20 causes of disability worldwide (10). The lost time and productivity in the workplace, the costs of medical care and inpatient treatments, makes migraine the most costly, painful, disabling condition in the US (11). These factors may contribute to the increased prevalence of migraine in the US among those of color and those facing economic hardship.

Our treatments for migraines have progressed since the application of leeches and compressed letting of blood. Advances in therapeutics have progressed from the early insights related to the efficacy of the ergot alkaloids, though the implementation of the tricyclic antidepressants, anti-epileptics, beta-blockers, calcium channel antagonists, and non-steroidal anti-inflammatory drugs which were the mainstay of migraine treatment from the ‘60s through the better part of the ‘90s and into the 2000s. Implementation of many of these therapeutics were rationally predicated on the evidence that migraines had a strong intracranial vascular component that served to activate the intracranial innervation of the meninges. I would like to consider several advances based on mechanisms.

## Regulation of Trigeminovascular Afferent Activity

Ligand receptor modeling brought us the triptans and an appreciation of the role of serotonin receptor subtypes which regulated the excitability of the meningeal trigeminovascular meningeal afferent terminals (12). In this regard, not all therapeutics arise from a rational focus. The ancillary observation of the use of botulinum toxins for cosmetic purposes led to the observation of a reduced incidence of attacks in migraineurs. While controversial, it soon became appreciated that the efficacy of this approach was in part dependent upon the “chronic” characteristics of the migraine phenotype (13), an observation reminding us that all migraines are not the same. Since the mid-1990s, I have been intrigued by onabotulinumtoxin A in migraine headache disorders. We knew so little then, other than on its effects on the SNAP25 protein essential for mobilizing synaptic vesicles (14). Questions arose: How could muscle tension relief prevent migraine? Little did I suspect that something we observed in the biochemistry lab of the movement of proteins in the axonal medium would be responsible for the observed effects of subcutaneous tissue of the cranium. Here, it is clear that these molecules are taken up and transported centrally to the trigeminal ganglion and thence to the terminals in the nucleus caudalis where, though transneuronal movement, the input from the meningeal trigeminovascular afferents could be mitigated (15, 16). Still, we learn more from that “continual and fearless sifting and winnowing” of knowledge (attributed to Charles Kendall Adams) to find potential new uses for this complex molecule (17). Future directions will doubtless focus on this regulation and may further our insights on these mechanisms.

### Role of Local Meningeal Transmitters

The discovery that calcitonin gene-related peptide (CGRP), released from the afferent terminal innervating the meninges and known to result in meningeal vessel dilation and plasma extravasation, was significantly elevated in venous out flow during a migraine attack in the 1980s led to “anti-CGRP” with antibodies and molecules targeting the receptor or the peptide itself. These new oral medications, called “gepants,” represented engagement of a truly novel target based on our basic understanding of the trigeminovascular system (18). Other targets associated with this system are on the horizon, such as those targeting cortical/afferent vasoactive peptides such as PACAP and VIP (19).

These treatment advances, resulting in efficacy to the treatment of migraine and a marked improvement in patients' quality of life, were enabled by the evolving understanding of the systems involved in the merger of detailed insights evolving from elegant preclinical work with clinical study. While the human migraine experience is clearly complex and likely subject to interspecies differences, the parallels between events that define the human migraine phenotype, and the pharmacological physiological and behavioral characteristics of the preclinical models, emphasize their relevance to our understanding of the migraine phenotype in human. I would note several pertinent mechanisms, old and new, that should engage our attention.

## Spreading Depression

Arguably the original preclinical model for migraine arose from the work of Leao (20), who was looking for a better understanding of epilepsy using rabbit, pigeon, and feline models. However, he later drew the first connections between this and migraine based on the nature of the aura reported by the migraineur. That initial work has led to the investigation of and literally thousands of studies on the nature of cortical spreading depression (CSD). Studies have examined how, when, where, and why it leads to advances in understanding the migraine process. This work has translated down over time to identify the role of excitatory neurotransmitters, such as glutamate (21), and potentially the effects of disruption of blood-brain barrier function (22). The mode has provided an important system platform to assess the role of various processes that may be associated with migraine headaches, such as genetic modulators, the role of estrogenic and other hormonal factors, and the physiological consequences of stress.

Life events such as sleep deprivation can be induced in animal models leading to increased occurrence of CSD (23). Questions must be raised that have not been answered, such as why ischemic stroke (24) provokes CSD but not a migraine-like headache. With the blood-brain barrier being a significant obstacle to drugs to block spreading depression itself, our increasing understanding of the downstream effects of CSD may allow us to target secondary changes in pain regulation, endothelial stability, and inflammatory molecules in the migraine process. This impact on inflammation in migraine has come back around to us after Wolff described a sterile inflammatory process associated with dural vessels in migraine attacks with ongoing studies of CSD impacting not just the upregulation of multiple inflammatory markers but specific ones (25) in those with a form of migraine with aura associated with hemiplegia.

## Glymphatics

Neuroanatomists have contributed to the relatively recent findings of the CNS glymphatic (26) since the early second decade of this century. Researchers have characterized the cell structure of these distributed flow channels associated with all the arterial circulation to the capillary level. Over the last decade, we have learned a great deal about its circadian function and impact on fluid management by the brain and how CSD (27) can interrupt this system. Technological advances in the lab are allowing us to use optogenetic methods to induce and monitor CSD (28, 29).

## Nonconventional Migraine Therapeutics

While natural and herbal therapies, such as the use of riboflavin (and historically almost all the B-vitamins), have a recent history in migraine treatment, their use may go back centuries. Hildegard, an abbess in Bingen Germany, had visions in which she had her nuns turned into tapestries. Throughout these are evidence of migraine aura events. The occurrence of ergotism was common in the area and period. The common rumor has been that the concentration of ergot was more significant on one side of the Rhine than the other. This led to vascular occlusion on one side and migraine-like events on the other. We do not know whether eating moldy black bread was the cause or cure of Hildgegaard's migraines. A host of poisons and healthy herbs were used over the centuries with some benefit. The turn of the 19th century found research on modifying the ergot compound into a medicinal agent. Among the agents obtained from this is lysergic acid diethylamide, which started its life in psychiatry and became the “drug” of the summer of love. It also led to ergotamine tartrate and mesylate being beneficial for migraine and other headache disorders by Wolff. The ability for the chemist to use advancing techniques of understanding of the conformational image of molecules such as these and to apply X-ray crystallography among the tools for modeling the receptors for them has led to the development of sumatriptan and is playing a role in the use of “hallucinogenic” agents as therapeutics in headache and mood disorders. Even non-hallucinogenics have been transforming headache and other medical disorders (30). I imagine that few of us who were around in the early days of the popularity of marijuana would have imagined us taking the chemical substrates into the lab. There we have begun to understand the potential for various related compounds to have beneficial effects in headache disorders in a complex fashion (31) and lead to potentially precise and well-tolerated treatments of migraine and chronic migraine.

## Advances in the Genomics of Migraine

The advances in technology and scientific application of genomic analysis technology have not been wasted in headache medicine and have pointed to novel mechanisms and potential interventions. We find increasing numbers of genetic loci potentially encoding (32) for migraine within the genome, but none have traversed even the modest advances on gene sequence. There are also strong migraine links to women (33) and suggestions of mitochondrial involvement (34) in the predilection for migraine. Additionally, we have only begun to touch the surface of our understanding of the role of selective nutritional factors that are important in the cellular process. Various neuronal (35), vascular (36), and inflammatory (37) mediating cell receptors are targets of neurotransmitters (38) and neuropeptides (39) involved in migraine. However, we have yet to discern the end effects of how these receptors lead to a migraine headache.

A widely quoted example is that of familial hemiplegic migraine. Exciting evidence points to the CACNA1A (FHM1), ATP1A2 (FHM2), and SCN1A (FHM3) (40) genes and their role in the expression of three different channels: a voltage-dependent PQ calcium channel, a sodium/potassium ATPase, and a sodium channel. These insights may allow us to learn not only how these mutations lead to hemiplegia but the associated migraine as well. This work raises many relevant issues. Where are these channels located? Are they in the cortex? Are they distributed uniformly or in preferential areas such as the motor cortex? Are they in the neurons themselves or glial or other supportive cells? Does the alteration in cell function associated with these channels being altered interplay with other genetic factors to bring on the attacks, or are they sufficient in themselves? Regardless, it is crucial to wonder why they are not impacting the patients with this gene variant continuously but rather on rare occasions. Is there evidence of them existing solely within the blood barrier, or may they be in the endothelium or muscular layers of arteries responsible for long-term consequences of persistent neurological defects? This, too, would impact our ability to target these receptors. It seems that only minuscule amounts of monoclonal antibodies traverse the blood-brain barrier and so leave only small molecule drugs as agents for treatment.

## Changes in Brain Connectivity and the Migraine Phenotype

Our knowledge of connectivity (41, 42) within the cortex through MEG and fMRI has helped us understand some of the clinical characteristics of headache disorders. However, it has not helped us find the “seat” of migraine nor understand the sequencing of events that occur and whether there is but one basis for migraine or whether migraine is a clinical syndrome with multiple underlying sources. Is migraine with aura genuinely arising from the cortex, or is it generated elsewhere (43, 44)? Is only the visual cortex (45) susceptible to generating cortical spreading depression? I queried Jes Olsen once on this (personal communication). He suggested that perhaps other areas could be the generator, such as in the temporal lobe, but that the symptoms produced by such an “aura” may be hard for the individual to recognize. Could migraine indeed have its generator in the periaqueductal gray matter? This site was suggested by observing the effects of sumatriptan to reverse the changes on fMRI in a migraine attack induced by CGRP infusion (46). Perhaps the hypothalamus is a responsible structure for headaches. Migraine prodromal symptoms are characteristic of hypothalamic activity (47), and the autonomic disturbances so characteristic of cluster headache appear to arise here as well.

## Advances in Neuroimaging

Our advances in animal and human neuroimaging and neurophysiology have served to spur developments allowing us to translate animal models to the human migraineur (and vice versa) and even to those with actively occurring attacks of headache disorders (48). Some of these advances over the years have led us down false pathways such as cerebral blood flow studies that seemed to support Wolff's hypotheses of a unique vascular basis for migraine. Advances in the technology of these measurements and coupling them to other modalities has led to corroboration of the work of Leao on cortical spreading depression and its relationship to the migraine aura, as classically suggested by Lashley (49). Similarly, advances in magnetic resonance imaging have come far since I was a college sophomore using an MRI progenitor to determine organic compounds' chemical structure to visualize organic moieties in the perivascular space with 7 Tesla MRI images (50, 51). Other headache disorders hopefully will benefit from the advances in these diagnostic modalities. Our boon of visualizing small vascular and neuronal structures has proven to significantly benefit many patients with craniofacial pain, such as trigeminal neuralgia, and the finding of vascular loops has made the disorder tratable, albeit with a significant surgical procedure. Questions linger, however, as to why this procedure, like other treatments, eventually fails. Or why those with the most “typical” form of Trigeminal Neuralgia are the most likely to have success (52). Being able to determine the occurrence of demyelination of nerve adjacent to the vascular loop or alteration in Trigeminal Nerve hyperactivity might greatly reduce the use of a surgical procedures more likely to fail based on objective evidence than the patient's history.

## The Microbiome

Exciting advances in studying the microbiome and the role played by these intrinsic occupants of our body has led to the appreciation that these molecular generators of circulating products may contribute to the headache phenotype. Studies showing a role for TLR4 signaling in preclinical studies are evocative (53). Might the gut biome serve as a modulator of disease expression? May it explain why no single medication or other treatment is effective in 100% of patients with a specific form of headache? The complexity of the science involved in dissecting the gut biome will not be simple, but the role of the biome in pain and various neurological disorders has shown significant covariates (54, 55).

## Novel Delivery Platforms

We have seen advances in delivery therapy to patients leading to improved clinical outcomes through improved compliance and tolerability with the extended-release formulation. It has, however, only been since the turn of the millennium that meaningful improvements have occurred. Improved delivery of a non-steroidal anti-inflammatory drug, diclofenac, occurred with a potassium salt combination (56) dissolved in a thimble full of water. This compound produced an equivalent response to sumatriptan tablets—this efficacy occurring in as little as 10 min. Sumatriptan itself has been delivered as a non-needle subcutaneous delivery using compressed gas (57), which, while effective, never really caught on. Nasal liquid sumatriptan has always been plagued by taste issues, poor absorption, and marginal efficacy. These were addressed first with a dry powder inhalation (58) and now with a liquid formulation with an adjunctive agent's addition. This change increased permeability (59) of the nasal mucosa, which has improved taste and led to more rapid responses and migraine pain relief approaching the subcutaneous injection. One transdermal preparation had a short life span because of burns associated with an iontophoretic delivery (60). Another new approach with zolmitriptan using a micro-needle array (61) delivers a fraction of the typical oral formulation. However, it appears to produce efficacy results superior to oral and with negligible adverse events. Even the old standby dihydroergotamine (62) has changed delivery. It went from intravenous to intramuscular to subcutaneous to a large volume nasal spray. It is emerging as an improved nasal spray liquid (63) and a dry powder formulation (64), both of which promise to be better tolerated and likely equal or more effective than the current spray and potentially offer a replacement for injectable routes.

## Cost Effectiveness Considerations

Unfortunately, these advances in newer therapeutic modalities and deliveries come with the actual cost (65) of the research, development, individuals, manufacturing, delivery, and marketing. This type of progress can make the products expensive, not easily affordable without insurance, and even associated with insurance (66) there remain significant cash outlays. The pharmaceutical industry first addressed this with sampling. Sampling is now often frowned upon. So, they have moved to patient assistance programs. However, many patients, either because of their income level or federally sponsored insurance, are precluded (67) from these patient assistance programs. This puts the “best” of treatments out of reach of these persons who in many respects are most in need of obtaining control on their migraines to restore productivity and quality of life.

## Clinical and Biological Markers of the Migraine Phenotype

We continue to search for biological markers and diagnostic tools to establish headache diagnoses. We have progressed in better categorizing patients' signs and symptoms into relatively reliable diagnostic guidelines. The guidelines developed by the international classification of headache disorders (68) has evolved since their origin in the late 1980s compared to the original National Institute of Neurological Disorders' approximately one-page classification of headache disorders (69) from the early 1960s. However, it is still a matter of obtaining adequate and accurate information from the patient by a competent and knowledgeable provider to coalesce the information, perform an appropriate examination, and proclaim a diagnosis. No diagnostic study can accurately diagnose primary headache disorders. Attempts at finding markers (70) in the blood (71, 72), cerebrospinal fluid (73), neuroimaging (42, 74, 75), and immunologic factor (76) have not proven adequate. Even when we are close (72, 77), such as measuring CGRP in jugular venous blood, the measurement itself becomes subject to debate within laboratories on the adequacy of the specific methods used to measure it. With better diagnosis and reliable biomarkers, we move closer to delivering precision medicine to our patients, allowing for earlier diagnosis and treatment and potential for avoiding chronification and treatment resistance that occurs too often today.

## Future of Headache Medicine

The future headache medicine frontiers holds for us is now more than a “pie in the sky” situation. Artificial intelligence may help give enough scenarios to analyze the historical, physical, environmental, and genetic factors related to treatments and natural history. However, this approach is still in its early stages. An exciting avenue of research that may hold substantial promise is our understanding of the gut biome (78). May we find the answers to explain why there are so many migraine variants and other headache disorders? What of the familial occurrence of migraine, or the role of the microbiome? To date, the history of headache and its treatment has been one of some level of science coupled with good fortune and serendipity. However, to explore new frontiers of headache medicine and take us where none have gone before requires dedication. We need dedicated study of the socio-economic, genetic, and environmental underpinnings of the disorder. Scientists need to explore new targets for its biology and examine ways to manipulate the biochemical factors to achieve new treatments. We need to pursue the application of technology to study migraine and other headaches non-invasively. This pursuit needs to be done in those with these disorders. It may use the rapidly expanding fields of machine learning (79) and artificial intelligence (80) to synthesize all these components with the patient's experiences to diagnose the patient accurately and identify the specific treatment, tailor-made for them to remit if not cure the disorder. It is in these areas of interest and research I wish to see Frontiers in Pain Research-Headache take us. We have a grand challenge; indeed, we have many grand challenges that lay before us. Though, we come from many fields with different interests and expertise, we need to engage and share our ideas to bring us to a newer and better place in the field of headache.

## Author Contributions

The author confirms being the sole contributor of this work and has approved it for publication.

## Conflict of Interest

The author declares that the research was conducted in the absence of any commercial or financial relationships that could be construed as a potential conflict of interest.

## Publisher's Note

All claims expressed in this article are solely those of the authors and do not necessarily represent those of their affiliated organizations, or those of the publisher, the editors and the reviewers. Any product that may be evaluated in this article, or claim that may be made by its manufacturer, is not guaranteed or endorsed by the publisher.

## Footnotes

^1^ημ*ι*κρᾱνíα (hēmikrānía), from ημι – (hēmi-, “hemi-, half”) + κρανíν (kraníon, “skull”).
